# High levels of caregiver burden in Prader-Willi syndrome

**DOI:** 10.1371/journal.pone.0194655

**Published:** 2018-03-26

**Authors:** Nathalie Kayadjanian, Lauren Schwartz, Evan Farrar, Katherine Anne Comtois, Theresa V. Strong

**Affiliations:** 1 Foundation for Prader-Willi Research, Walnut, California, United States of America; 2 PWS-Clinical Trial Consortium, Walnut, California, United States of America; 3 Department of Rehabilitation Medicine, University of Washington, Seattle, Washington, United States of America; 4 Prader-Willi Syndrome Association (USA), Sarasota, Florida, United States of America; 5 Department of Psychiatry and Behavioral Sciences, University of Washington, Seattle, Washington, United States of America; 6 Department of Genetics, University of Alabama, Birmingham, Alabama, United States of America; Universite de Bretagne Occidentale, FRANCE

## Abstract

**Objectives:**

Prader-Willi syndrome (PWS) is a rare genetic neurodevelopmental disorder that is characterized by hyperphagia, developmental delay, incomplete sexual development, mild-to-moderate intellectual disability, and a variety of challenging behavioral and psychiatric symptoms. The characteristics of PWS can be difficult for caregivers to cope with and are likely to cause significant and long- term caregiver burden. The current study examined burden in 142 caregivers of children and adults with PWS living in the US using the Zarit Burden Interview (ZBI). The study aimed to measure the level of burden in caregivers of individuals with PWS, to explore the impact of PWS on caregiver quality of life, and to assess ZBI as an indicator of that impact.

**Results:**

Caregivers participating in this study were predominantly mothers, 30–59 years old, non-Hispanic Whites, married or in a relationship, with an annual household income slightly distributed towards higher income. Nearly 90% of the caregiver`s children with PWS lived at home. Caregivers experienced high caregiver burden with an average ZBI score of 44.4 ± 15.4. ZBI scores were highest for caregivers of teenage and young adult individuals with PWS (49.2 ± 14.6 and 49.2 ± 14.1, respectively), while those caring for older adults (>30) and the youngest age group had lower scores (38.6 ±10.5 and 34.8 ±12.5, respectively). Caregivers reported that caring for a person with PWS negatively impacted their romantic relationship, ability to work, sleep, and mood. Whereas we did not find strong correlations between family income or level of help the caregiver receives and ZBI scores, the results showed significant correlations and a linear relationship between ZBI scores and caregiver depressed mood, feelings of anxiety, negative romantic relationship impact, as well as sleep and work disruption.

**Conclusions:**

Our study reveals that PWS incurs high caregiver burden and impacts many aspects of the lives of caregiver. We identified the ZBI as a good predictor of that impact. Our findings draw attention to the critical unmet need for support for caregivers of individuals with PWS.

## Introduction

Prader-Willi syndrome (PWS) is a rare genetic neurodevelopmental disorder that occurs in approximately 1 in 15,000 to 30,000 births [1] and is caused by an absence of paternally expressed, imprinted genes on chromosome 15q11–q13. Individuals with PWS present with hypotonia and feeding problems in early infancy, followed during childhood and adulthood by intellectual and learning disabilities, maladaptive and compulsive behaviors, incomplete sexual development, and severe hyperphagia. Behavioral challenges are often prominent in PWS and include temper tantrums, intractability, obsessive–compulsive and repetitive behaviors, aggression, oppositional behavior, and skin-picking [2, 3]. As individuals with PWS reach adulthood, they are at high risk for developing psychiatric illness, including psychosis and major depression [4]. Behavior related to food is also complex in PWS. There is a progression that occurs in PWS beginning with failure to thrive in infancy, moving to an increase in weight followed by an increasingly strong interest in food, and finally to severe hyperphagia associated with a lack of satiety [5]. Unless eating is strictly controlled externally, the overwhelming drive to eat coupled with reduced energy expenditure and decreased caloric requirements leads to morbid obesity. There is currently no cure for PWS. Growth hormone therapy is the only FDA-approved therapy for use in children with PWS. Despite beneficial effects on height, body composition, strength, endurance, bone mineral density, respiratory quotient and sense of well-being [6, 7] it has no discernable impact on hyperphagia or behavioral challenges. The severe hyperphagia, psychiatric symptoms and lack of treatments all create unique challenges in caring for persons with PWS.

Understanding the potential negative impact of caregiving for a special needs child is important because the burden and lack of wellbeing that caregivers experience can reach clinical levels for the caregiver and directly impacts the quality of care and the outcome for their vulnerable children with PWS [8]. Previous studies of PWS caregivers have indirectly assessed the burden of caregiving via examining parent/caregiver psychological distress, coping, and family/interpersonal relationships. When compared to families of children with a variety of causes of intellectual disability or complex health conditions, parents of children with PWS report higher levels of stress and mood disruption, more difficulties coping with their child’s symptoms [9, 10], higher levels of family conflict as well as lower quality of life [11, 12]. Taken together these studies suggest that PWS may impact parent caregiver mood, stress levels, and family relationships, to a greater degree than is seen in caregivers of children with other conditions.

Fulfillment of the caregiving role can also have significant impact upon the caregiver’s ability to work and the family’s financial situation. Parents of children with intellectual disabilities report more limited employment due to their child with special needs [13]. This curtailed employment can be associated with feelings of isolation, lack of fulfillment, and low self-esteem for the caregiver [14]. Families of children with special needs also often have multiple additional expenses not associated with raising a typically developing child [15]. A recent study performed in Europe showed that individuals with PWS incur considerable healthcare costs [16]. It is not known, however, whether caregiver employment and family economic health affect the perception of burden in PWS.

Sleep quantity and quality is an important aspect of well-being and has been shown to be highly related to overall quality of life [17]. Greater sleep problems are found in caregivers of children with special needs compared to parents of typically developing children [18]. To date, no study has examined sleep in PWS caregivers and the potential impacts on caregiver quality of life. It should be noted that people with PWS also struggle with significant sleep challenges including apnea, early waking and excessive daytime sleepiness [19] which may adversely impact the caregiver’s sleep.

Given the multiple, life-long and complex challenges inherent in PWS, it is likely that PWS impacts significantly many aspects of caregiver quality of life. One way to understand these impacts is through the lens of caregiver burden, which is defined broadly as feelings of stress, embarrassment, guilt, resentment, isolation and loss of control [20]. In the present study, we first aimed to directly measure caregiver burden in PWS using the Zarit Burden Interview (ZBI) [21]. The ZBI is the most frequently used instrument to assess the psychosocial and health toll of caregiving an individual with a disability or chronic illness. Second, we assessed the impact of PWS on aspects of caregiver life using a specific questionnaire about the relationship between PWS and work, finances, caregiver mood symptoms and sleep, and impact on marital/romantic relationship. Finally, we examined whether the ZBI predicts that impact.

## Methods

### Participants

PWS caregivers were recruited from various PWS Facebook pages sponsored by the two primary US PWS advocacy groups, the Foundation for Prader-Willi Research (FPWR) and the Prader-Willi Syndrome Association (USA) (PWSA-USA). One hundred and ninety-four (194) PWS caregivers completed the survey. One hundred and fifty-two (152) of those caregiver respondents identified the United States as their country of residence and were included in this study. Of those, ten (10) had incomplete ZBI data and were not included, resulting in a total of 142 respondent survey data used for the analyses.

### Assessment

Caregiver burden in PWS was evaluated using an online, anonymous self-reported PWS Caregiver Survey comprising of 59 questions including individuals with PWS and caregiver demographics, the ZBI and additional questions related to the specific impact of PWS on many aspects of caregiver life. The term “child” was used throughout the survey to describe the person with PWS for whom care was provided.

The ZBI (Copyright 1980, 1983, 1990 Steven H Zarit and Judy M Zarit) is a 22-item self-report questionnaire completed by caregivers who were asked to rate their experiences on a 5-point Likert scale where 0 = never and 4 = nearly always. The 22-item ZBI encompasses questions related to caregiver health and psychological wellbeing, finances, impact on social life, and relationship with the individual with PWS. Responses were used to derive the ZBI total score (score range 0–88), where higher scores represent greater burden. A global ZBI score was calculated for each participant consisting of the sum of all the caregiver ratings ranging from “0” (no burden) to “88” (highest burden). The measure has been shown to have good internal consistency, content validity, and test-retest reliability [22], The range of scores from 21 to 40 are considered mild-to-moderate burden [20], and scores equal or above 41 considered high to severe burden.

As part of the online PWS Caregiver Survey, 37 self-report questions were included to gather demographic information and evaluate the impact of PWS on various aspects of the caregiver’s quality of life, which encompassed questions about work, finances, mental and physical health, and social relationships/support. Responses were collected using multiple-choice, visual analogue and Likert-scales. As part of this survey, Caregivers were asked to rate four questions on a 1 to 10 Visual Analogue Scale (VAS) [23] (1 = Not at all and 10 = Extremely): 1) How anxious or worried have you felt over the last 2 weeks; 2) How depressed, sad or hopeless have you felt over the last 2 weeks; 3) How much help do you have with caring for your child with PWS (family, paid caregiver, friends) and 4) Rate the degree to which having a child with PWS has adversely affected your marriage/romantic relationships. The Survey also included questions about the impact of PWS on caregiver employment: 1) I made changes to my work related to my child with PWS (yes/no); 2) I miss work or need to alter my work schedule related to my child with PWS (never; rarely (1–2 times a year); occasionally (once a month); frequently (several times per month)); and 3) my child’s other parent has made changes to their work related to our child with PWS (yes/no). PWS was considered to have an impact on work if the caregiver or child`s other parent made changes to their work or if their work had been disrupted at least once/month. To assess the impact of PWS on caregiver sleep, caregivers were asked to respond by Yes or No to the question “I get less sleep because of my child with PWS”.

### Procedures and ethical statement

The online survey was developed using Survey Monkey software and pre-tested in a sample of 10 PWS caregivers before it was open to all PWS caregivers. The survey took about 15–20 minutes to be completed. Anonymous data were collected between May 10, 2016 and June 10, 2016. The study was approved by the Hummingbird IRB committee (#2017–57).

### Statistical analyses

Descriptive statistics are reported as percentages and mean±SD. Spearman correlation coefficients were used to examine relationships between variables. The age of the person with PWS was converted to 5 age groups 0–4, 5–11, 12–18, 19–30 and 31 years and above. The age breakdown aligns generally with the progression of the disorder, with early years (age 0–4) characterized by decreased or normal appetite; childhood years (5–11) characterized by increased interest in food and associated behaviors; adolescence (12–18) and young adulthood (19–30) characterized by hyperphagia and increased behavioral and mental health challenges, and older years associated with moderation of behaviors in some individuals [5, 24]. A one-way between subjects ANOVA was conducted to compare the association of child’s age group and caregiver burden. When the association was found significant, post hoc comparisons were performed using the Tukey HSD. Analyses of relationships between the continuous ZBI scores and categorical variables (impact of PWS on work and sleep) involved ANOVA test. Linear regression was used to test if caregiver burden (total ZBI score) predicts impact of PWS on caregiver mood (feelings of anxiety and depressed low or mood), and romantic relationships. Logistical regression was used to assess whether caregiver burden predicted a negative impact of PWS on caregiver work and sleep. Univariate relationships of potentially confounding demographic variables (age, ethnicity, marital status, income level, and whether the child lived at home full time) were examined with both the independent and dependent variables. Only one variable (whether the child lived at home or with other family versus living outside the home) met this standard for the outcome measures of low mood, feelings of anxiety, and sleep and was therefore included in the regression analyses for these outcomes. Statistical significance was set at α = 0.05. SPSS Statistics 19 was used for analysis of results.

## Results

### Caregivers

The caregiver group (n = 142) was predominantly composed of mothers, followed by fathers, relatives (e.g. sibling, other relatives) and non-relative guardians (Table 1). The age of caregivers was distributed across all age categories with 72% of them aged between 30 and 59 years. Non-Hispanic whites constituted the majority of the caregivers. Most respondents were married or in a relationship. The annual household income distribution was shifted towards higher income with 61.3% of caregivers reporting an income higher than $60,000 per year including 30.3% having an income greater or equal to $100,000 yearly (Table 1). More than 85% of caregivers reported receiving some help with caring for the person with PWS, including 17% receiving “a lot” of help (score of 10 on the 10-point VAS) (https://osf.io/mt3d5/?view_only=2b26d69294db4582901935804e26484b).

**Table 1:** Sociodemographic characteristics of participating PWS caregivers and individuals with PWS living in the US.

Variable	N (%)	Variable	N (%)
**Caregivers**		** **	
RelationshipwithChild		Ethnicity	
Mother	117 (82.4)	Asian	6 (4.2)
Father	12 (8.4)	Caucasian/ White non-Hispanic	114 (80.6)
Relatives	11 (7.8)	Black / African American	3 (2.1)
Non-relative guardians	2 (1.4)	Hispanic/ Latino	13 (9.2)
Age(years)		Other	4 (2.8)
20–29	8 (6)	Decline to answer	2 (1.4)
30–39	38 (27)	Annualhouseholdincome	
40–49	35 (25)	Under $9,000	3 (2.1)
50–59	39 (27)	$9,000 - $11,999	5 (3.5)
60–69	16 (11)	$12,000 - $19,999	8 (5.6)
70–79	3 (2)	$20,000 - $39,999	18 (12.7)
80+	2 (1)	$40,000 - $59,999	12 (8.5)
unknown	1 (1)	$60,000 - $79,999	21 (14.8)
Maritalstatus		$80,000 - $99,999	23 (16.2)
Married	104 (73.2)	$100,000 or over	43 (30.3)
Living together with partner	7 (5)	Decline to answer	9 (6.3)
Divorced	15 (10.6)		
Separated	4 (2.8)		
Widow	3 (2.1)		
Single	9 (6.3)		
**Individuals with PWS**			
Age(years)			
0–4	25 (17.6)		
5–11	33 (23.2)		
12–18	38 (26.8)		
19–30	34 (23.9)		
31+	12 (8.4)		
Livingsituations			
In your home (full time) or with other family	125 (88.0)		
On own	5 (3.5)		
Group home or other facility	10 (7.0)		
Other	2 (1.4)		

We found that the sociodemographic characteristics of the caregivers excluded from the analyses due to incomplete responses (n = 10) did not differ from those included in this analysis except for a higher representation of caregivers aged between 70 and 79 years (+10%) (https://osf.io/mt3d5/?view_only=2b26d69294db4582901935804e26484b).

### Individuals with PWS

The age of the individuals with PWS was relatively well distributed across infants to early adulthood, with the exception of individuals aged 31 years and older who were comparatively under-represented (Table 1). Most individuals with PWS lived at home with the caregiver or a family member whereas a minority of individuals lived in group homes and other facilities or on their own. The remaining 1.4% represented two individuals including an individual who had passed away at the time of the survey and one individual for whom the living situation information was left blank (Table 1).

We found that the sociodemographic characteristics of the individuals with PWS excluded from the analyses (n = 10) did not differ from those included in this analysis except for a higher representation of individuals aged above 31 years (40%) and a higher proportion of individuals living in group home or other facility (30%) (https://osf.io/mt3d5/?view_only=2b26d69294db4582901935804e26484b).

### Zarit burden Interview (ZBI)

**Fig 1:**
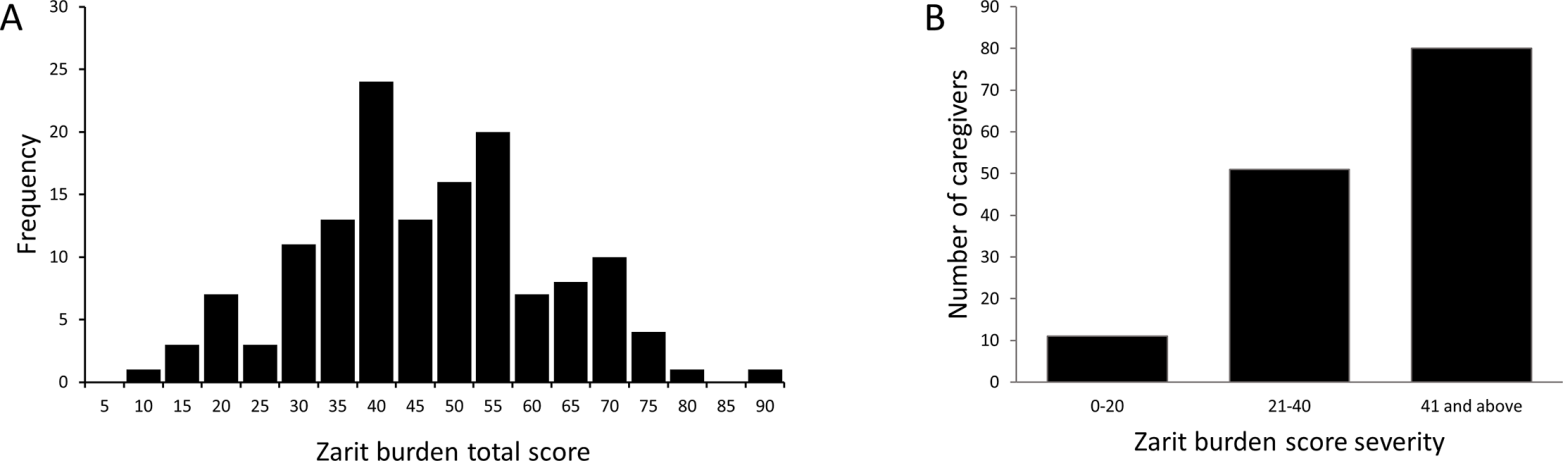
Zarit burden scores for caregivers of individuals with PWS. A. Frequency distribution of ZBI total scores in caregivers for PWS. B. Number of caregivers per category of burden severity: Mild (0–20), Moderate (21–40), High (41 and above).

The overall mean (SD) ZBI total score was 44.4 (15.4) and the median score was 43. The ZBI scores had a bell-shaped distribution with scores ranging from 6 to 86 (Fig 1A). Most caregivers (56%), reported a high level of burden, with a ZBI score above 40, while 36% of caregivers rated their burden in the mild to moderate range (ZBI scores ranging between 21 and 40) and 8% reported little to no burden (Fig 1B).

**Fig 2:**
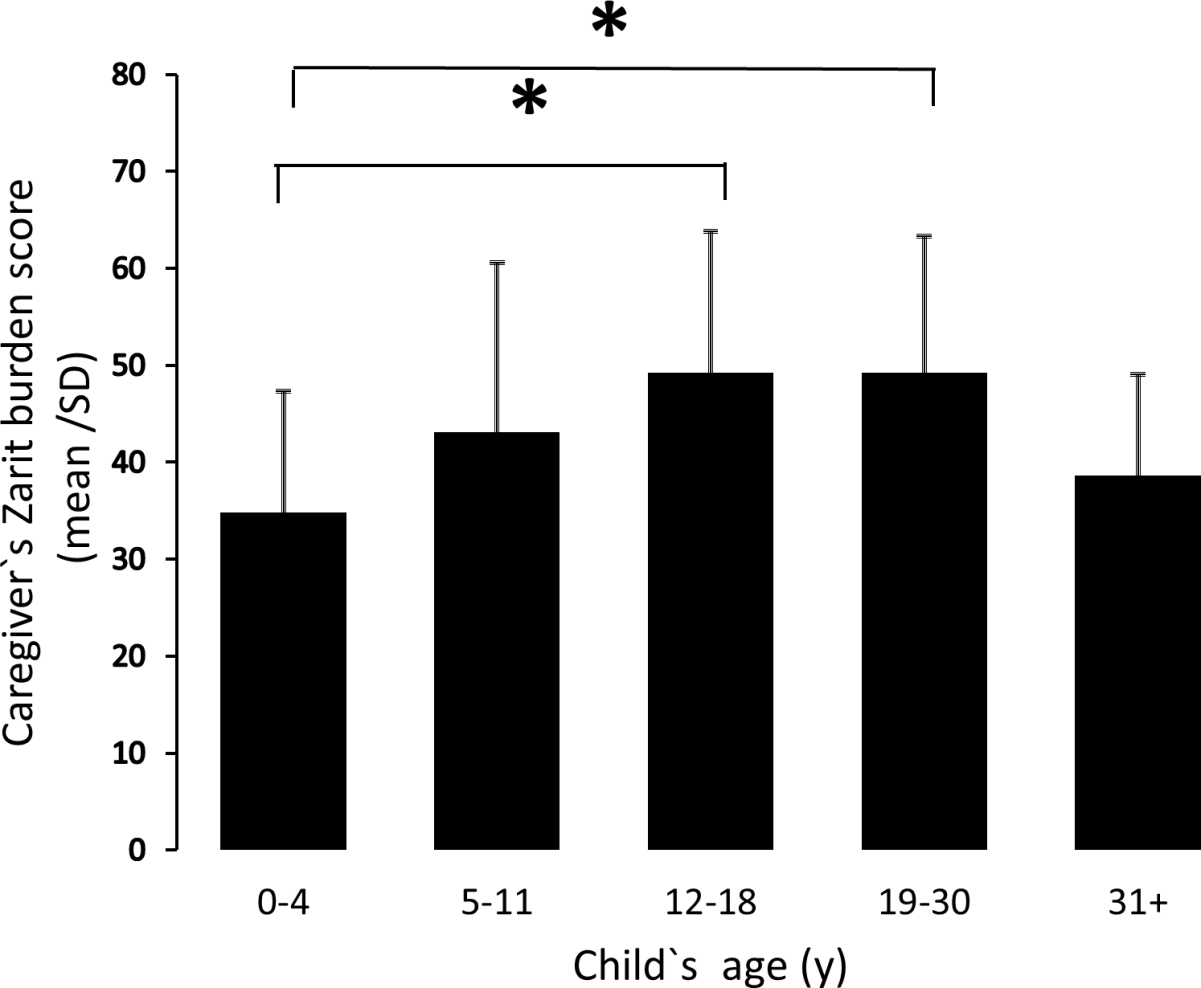
Caregiver Zarit burden scores vary with child`s age. Results are expressed as the caregiver`s Zarit burden mean score ± SD in function of the child`s age category (years). *P < 0.05 when compared to the 0–4 y category.

Caregiver burden varied significantly across the 5 child age groups (F (4,137) = 5.21 p < .001). Post hoc comparisons using the Tukey HSD test indicated that the ZBI score for caregivers of individuals in the 12–18 years (score: 49.2 ±14.6) and 19–30 years (score: 49.2 ±14.1) category were significantly higher than the 0–4 y age category (score: 34.8 ±12.5) (p < 0.05 for both 12–18 y and 19–30 y age category when compared to 0–4 y age category). In contrast, the mean ±SD ZBI score for caregivers of children aged 5–11 years (score: 43.1 ±17.5) and adults aged 31 years or more (score: 38.6 ±10.5) did not significantly differ from the 0–4 y age category (Fig 2). Taken together, these results suggest that the level of caregiver burden is the lowest for caregivers of infants and young children aged 0–4 years. Level of burden increases in caregivers of children aged 5–11 years and reaches its highest level in caregivers of adolescents and young adults. Caregiver burden tends to decrease in caregivers of adults aged 31 or above to levels similar to those caring for the youngest PWS group.

**Fig 3:**
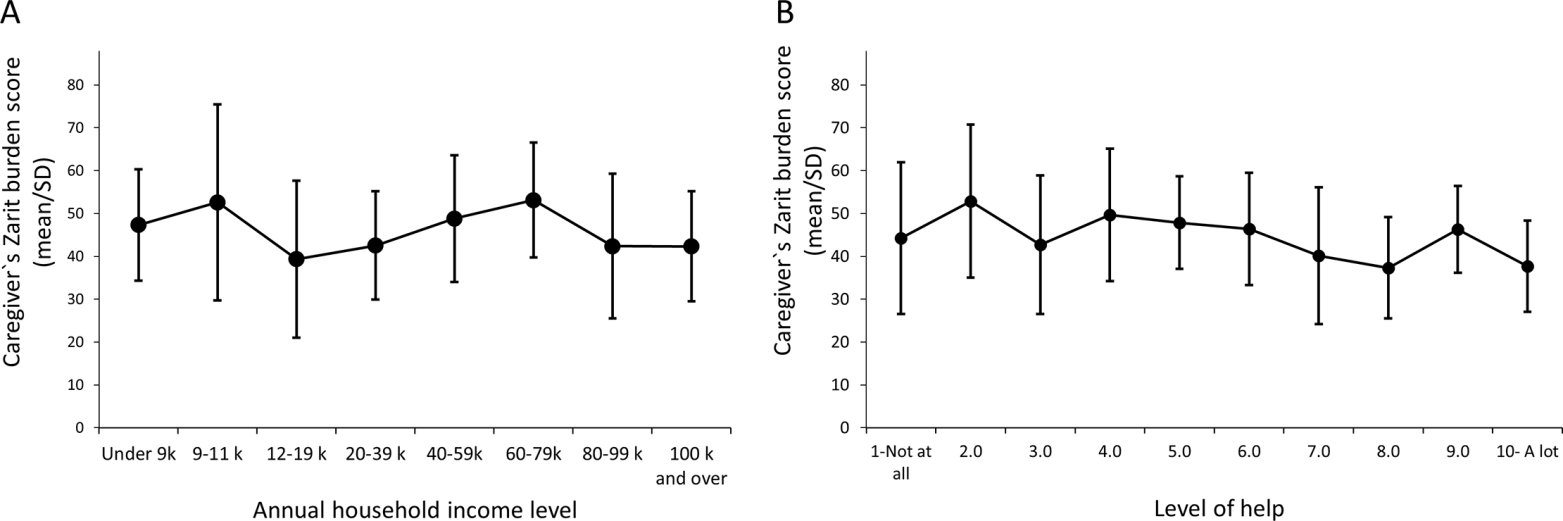
Caregiver Zarit burden scores are independent of the annual household income and the level of external help. Results are expressed as the caregiver`s Zarit burden mean score ± SD in function of the annual household income (A) or level of help expressed on a VAS (B).

A Pearson correlation coefficient was computed to assess the relationship between caregiver burden and the household income and the reported level of help received. Caregiver burden was not correlated with household income levels (r = -0.08, n = 133, p > 0.05). The mean Zarit burden scores were high across the range of annual household income, varying between a minimum of 39.4 (for an income between $12,000-$19,000) and a maximum of 52.6 (for an income between $60,000–79,000) (Fig 3A). Caregiver burden was weakly, although significantly correlated with the level of help for their child with PWS that caregivers reported receiving from family, paid care providers, and other persons (r = -0.24, n = 137, p < 0.05) (Fig 3B).

Mean ZBI scores were examined for differences between the individuals with PWS living at home or with other family (mean age: 12.9 ± 8.7 years, n = 125) versus those living outside the home or other family (ie group home, other facility or on their own; mean age: 32.1 ± 8.2 years, n = 15). There was a significant difference between groups (t = -2.75 p < .05) with mean ZBI score of 44.63 ± 1.39 versus 37.80 ± 2.06 for those living at home and those living outside the home, respectively.

### PWS impact on caregiver quality of life

**Fig 4:**
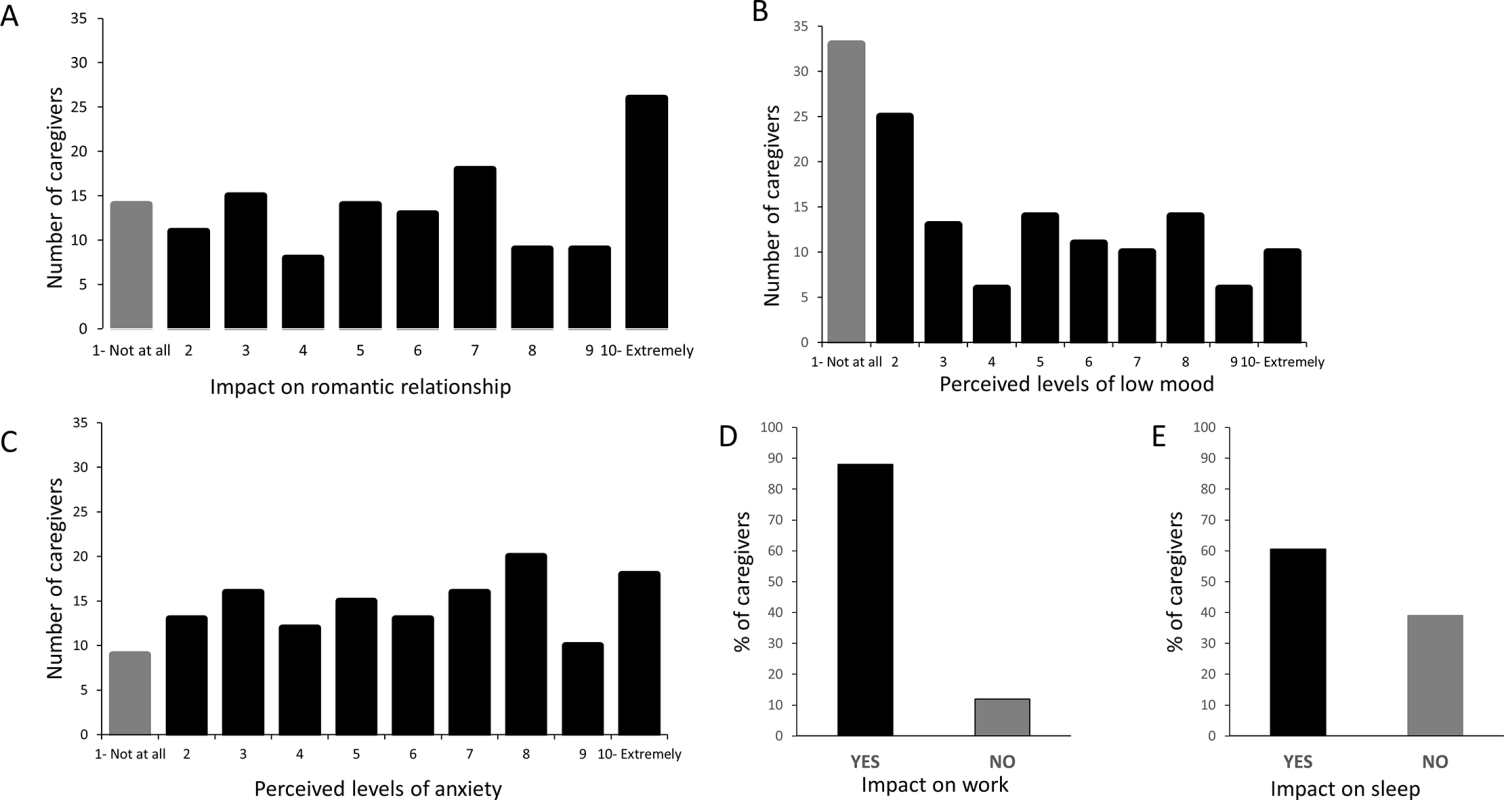
PWS impact on caregiver romantic relationship, perceived level of low mood, anxiety feelings, work and sleep. The number of caregivers is expressed in function of the level of impact on relationship (A), low mood (B), anxiety (C). Percentage of caregivers is expressed in function of the impact on work (D) and sleep (E).

Most caregivers (89.8%) reported that caring for an individual with PWS had an adverse impact on their marriage or romantic relationships, with more than half (55%) reporting a moderately high to extremely high negative impact on their relationship (score between 6 and 10 on the 10-point VAS) (Fig 4A).

Caregivers were also asked to report how depressed or hopeless they have felt over the last 2 weeks. Whereas 77% of caregivers reported at least some mild feelings of depressed or low mood, 35% of the caregivers reported scores reflecting moderately to extremely depressed or low mood (scores between 6 to 10 on the 10-point VAS) (Fig 4B). Caregivers were asked to report how anxious or worried they have felt over the last 2 weeks. Ninety-four percent of caregivers reported at least mild feelings of anxiety or worry, with 54% of caregivers reporting moderately high to extreme anxiety (scores between 6 and 10) (Fig 4C). Reported feelings of anxiety was strongly, positively and significantly correlated with reported feelings of depressed or low mood (r = 0.76, n = 142, p < 0.001) and was also positively and significantly correlated with the negative impact on the caregiver’s romantic relationship (r = 0.45, n = 137, p < 0.001). Most caregivers reported working full-time (43%) or part-time (21%). Of caregivers who worked, most (88%) reported that they or their partner had made changes to their work situation or altered their work schedule at least once a month due to PWS (Fig 4D). The majority of caregivers (61%) reported less sleep because of the person with PWS for whom they provided care (Fig 4E). Altogether, these results indicate that PWS has a significant impact on many aspects of caregiver quality of life, affecting their romantic relationships, their emotional well-being, and their ability to work and sleep.

### ZBI score is a good predictor of PWS impact on caregiver quality of life

**Fig 5:**
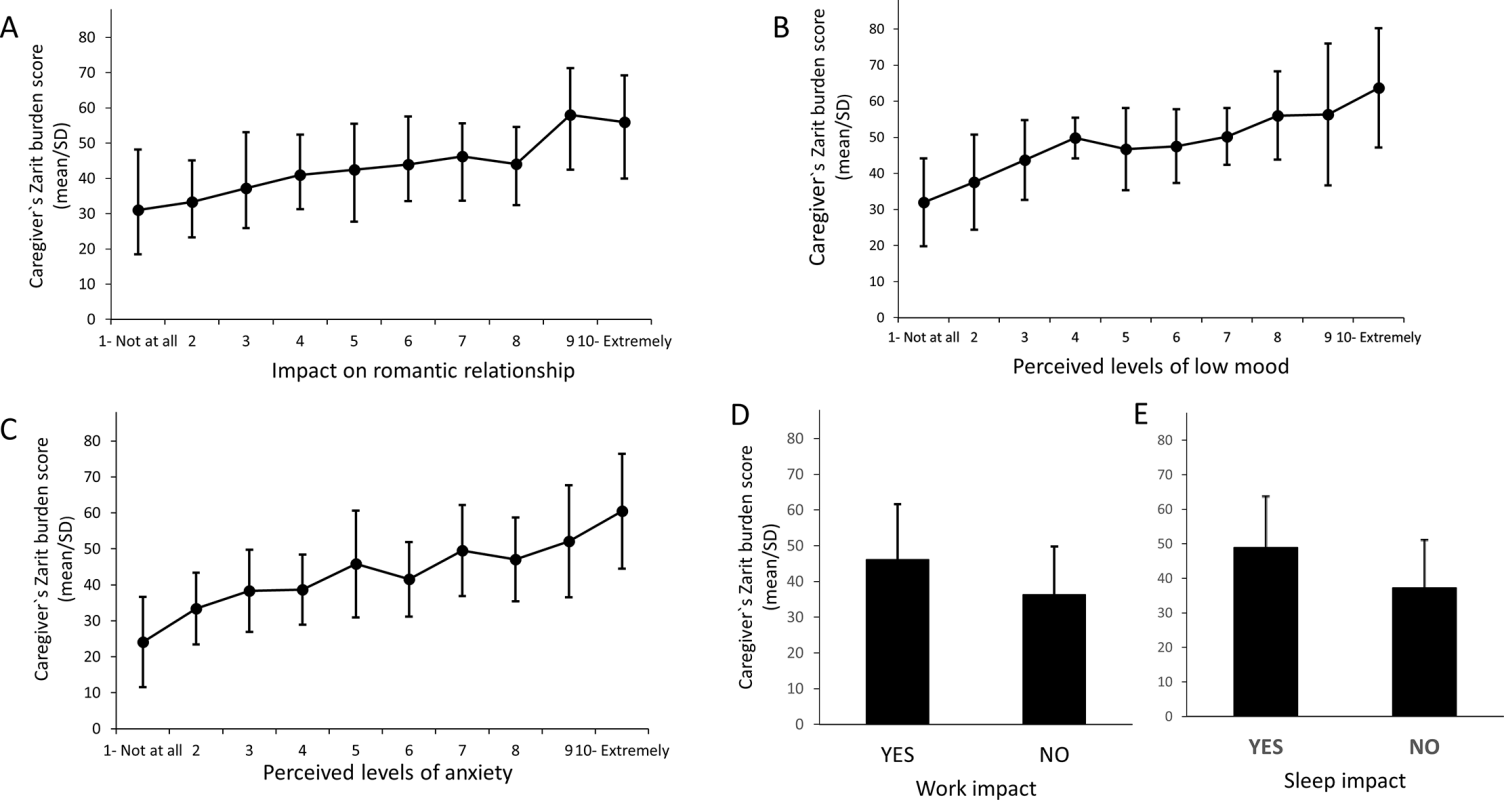
**Relationship between ZBI score and PWS impact on caregiver romantic relationship (A), perceived level of low mood (B), anxiety (C), work (D) and sleep (E).** The caregiver`s Zarit burden mean score ± SD is expressed in function of the impact on relationship (A), depressed /low mood (B), perceived level of anxiety (C), work impact (D) and sleep impact (E).

The Zarit burden scores correlated strongly, positively and significantly with the impact of PWS on the caregiver’s marriage or romantic relationships (r = 0.54; n = 137, p < 0.001), low or depressed mood (r = 0.61; n = 142, p < 0.001) and the perceived level of anxiety (r = 0.57; n = 142, p < 0.001) (Fig 5A–5C). In addition, the Zarit burden mean (SD) scores were significantly higher in those whose work had been affected by PWS (ZBI score: 46.2 ±15.4) as compared to those who were not affected (ZBI score: 36.3 ±13.6 t = 2.59; p < 0.05) (Fig 5D). Similarly, the Zarit burden mean ±SD scores were also higher in caregivers who reported getting less sleep because of the person with PWS (ZBI score: 48.9 ±14.8 versus 37.3 ±13.7 in caregivers whose sleep was not impacted) (t = 4.70; p < 0.001) (Fig 5E).

Linear regression was used to assess if ZBI scores predict the impact of caring for a person with PWS on caregiver romantic relationship, depressed /low mood, and feelings of anxiety. Caregiver ZBI scores predicted PWS negative impact on romantic relationship. A significant regression equation was found (F (1,135) = 54.79, p < 0.001) with an R^2^ of 0.29. For every 10-point increase in caregiver burden, a 1.1 (B coefficient = 0.11) decrease on the romantic relationship satisfaction VAS could be seen. Caregiver ZBI scores predicted depressed or low mood, adjusting for whether the person with PWS lived at home/ with other family or outside the home, (F(2,139) = 40.39, p < 0.001) with an R^2^ of 0.37. For every 10-point increase in caregiver burden, a 1.1 (B coefficient = 0.14) increase on the depressed mood VAS was calculated. Caregiver ZBI scores also predicted reported feelings of anxiety where a significant regression equation was revealed (F(2, 139) = 30.80, p < 0.001) with an R^2^ of 0.31. Thus, for every 10-point increase in caregiver burden, a 1.0 (B coefficient = 0.13) increase on the anxiety VAS was calculated.

Logistical regression was used to assess if ZBI scores predicted the impact of caring for a person with PWS on caregiver work and sleep. The odds of PWS having impacted work are one and a half times higher for every 10 point increase in caregiver burden (OR = 1.55, 95% CI = 1.13–2.11). The odds of PWS having impacted sleep (adjusted for whether the child lives with the caregiver or other family versus whether the child lives outside the family home) are almost twice as high for every 10 point increase in caregiver burden (OR = 1.69, 95% CI = 1.30–2.23).

## Discussion

The present study explored caregiver burden along with factors likely to impact the quality of life and experience of burden in caregivers for individuals with PWS. This study showed that caregiver burden as measured by the ZBI is strikingly high. Further, the reported burden appeared to be independent of income level and was only weakly correlated to the level of assistance that the caregiver reported receiving in caring for their child. Caregivers also reported negative impacts of caring for someone with PWS on aspects of their lives such as their romantic relationship, mood, anxiety, and ability to work and sleep. This is the first study to directly examine the relationship of the caregiver burden in PWS in relation to other aspects of quality of life for the caregiver. The present study demonstrated that the ZBI, a scale originally developed for carers of people with dementia, is pertinent to caregivers of individuals with PWS and is a good predictor of the impact of PWS on many aspects of the caregiver quality of life.

The caregivers for the individuals with PWS in our sample resided in the United States, and the large majority were married, non-Hispanic White mothers, aged between 30 and 59 years. The household income was distributed across all income ranges although it was slightly skewed towards higher income. The individuals with PWS were distributed homogenously across all age categories between birth and 30 years of age. Individuals aged 31 years and older were under-represented in our sample, which might reflect our online recruitment strategy [25] or the shorter life expectancy of those with PWS [26]. The majority of the individuals with PWS in our sample were living at home with the parent. This result is not surprising given that individuals with PWS require highly specialized care and supervision throughout their lives and other options are limited.

Of importance, the mean ZBI scores in PWS caregivers were higher than those measured in caregivers for persons with dementia, Alzheimer’s, and traumatic brain injury, all conditions known to have high levels of behavioral challenges in the individuals and to be difficult for caregivers [27–29]. There have been two recent studies using the ZBI in autism spectrum disorder, which found similarly high levels of caregiver burden as seen in the present study [30, 31]. One other study has examined the burden of PWS in caregivers living in Europe and found similarly high ZBI scores in some of the countries such as Spain, the UK and France, while lower scores were found in Germany and Sweden [16]. Although we cannot explain the score differences between countries, it may be possible that the age distribution for individuals with PWS differ between samples. Further studies examining caregiver burden as a function of the age of individuals with PWS across countries should be considered as a future line of research. The higher burden scores seen in those caring for individuals with PWS or autism may reflect differences between caring for an adult with an acquired injury or illness occurring later in life as compared to the challenges and difficulties caring for a child or adult with a lifelong neurodevelopmental condition.

Surprisingly, caregiver burden in PWS appeared to be independent of the level of income and was only weakly correlated to the amount of help caregivers reported receiving for caring for the person with PWS. Despite the income of the sample being somewhat skewed towards higher levels, the level of burden was high even in the highest income category. We also found that the level of caregiver burden was not related to how much help caregivers reported receiving. The quality and skill of the help received may be an important mediating factor given the unique behavioral challenges and hyperphagia symptoms seen in PWS. A survey of caregivers for early onset dementia found that it was very important for help to be tailored to their particular situation [32]. Given the multifactorial nature of caregiver burden, it is likely that dimensions other than costs and levels of help play an important role in caregiver burden in PWS.

The high level of caregiver burden in PWS could be due in part to the complexity and severity of symptom characteristics of individuals with PWS. Child behavior problems have been found to be the single most important predictor of caregiver psychological well-being [33]. A high risk for maladaptive behaviors and psychiatric problems is not unique to PWS, but these factors are more prevalent in PWS than in many other neurodevelopmental disorders associated with intellectual disabilities [34, 35]. The symptoms of hyperphagia seen in this population have been recognized as an extremely stressful and potentially life-threatening feature of PWS requiring constant vigilance on the part of the caregiver [36, 37]. Hyperphagia typically starts between ages 5 to 13 years and remains an issue throughout adolescence and adulthood. Additionally, greater behavioral and psychiatric problems are seen as the person with PWS ages, especially during adolescence and young adulthood [24, 35, 38–41]. The timing of increased hyperphagia and psychiatric/behavioral symptoms in PWS could explain the present results showing that caregiver burden in PWS reaches its highest level during the teen and younger adult years. As well, teenage and young adult years in the typical population are characterized by increased independence and development of a life independent from the caregiver. Given the intellectual disability and behavioral challenges in PWS, achieving these milestones may require considerable effort on the part of the caregiver, or may not be attainable at all. Research exploring the relationship between the individual`s levels of hyperphagia, psychiatric symptoms, intellectual disability, and caregiver burden will be important to understand whether these symptoms and their progression over time account for the high levels of caregiver burden seen in PWS. Living arrangement was also a significant variable with respect to caregiver burden, although additional studies are needed to determine if the relatively lower caregiver burden scores in the ‘living outside the home’ group reflects a moderation of symptoms in older individuals with PWS, decreased caregiving responsibilities for that living arrangement, or both. Regardless, caregiver burden in both groups is moderate to high.

The present study revealed that PWS has considerable impact on almost all major aspects of caregiver quality of life, including ability to work, emotional well-being, and sleep, which is consistent with previous research exploring potential impacts of special needs caregiving [9, 10, 42]. Additionally, caring for a person with PWS appears to also negatively impact the caregiver’s primary romantic relationship. Even though these aspects of quality of life were assessed with a single question each, the levels of feelings of anxiety, low mood and negative marital relationship impact reported in this non-clinical sample were notable. If cut off scores (ZBI between 24–26) for further mental health assessment as suggested by Schreiner and colleagues [43] were applied, a large number of caregivers in the present study would be identified as in need of further assessment and potentially at risk for developing clinically significant mental health symptoms.

Our study showed that the ZBI is a good predictor of the impact of PWS on many aspects of the caregiver quality of life and thus could be considered an efficient instrument to capture the more global impact of PWS on the primary caregiver. In addition, the present suggests that the ZBI might be a useful measure to assess interventions for caregivers to not only enhance their roles in the care of the person with PWS but also to focus on their personal well-being. Moreover, caregiver burden could be used as a supportive secondary endpoint for clinical trials focused on treating PWS symptoms. In fact, previous studies have shown that caregiver burden is reduced when symptoms of dementia are treated pharmacologically [44, 45]. Thus, the ZBI could be used as an outcome measure to assess effectiveness of treatments on individuals with PWS and their caregivers.

A number of limitations are present in the current study. These include its cross-sectional design, which suggests caution in the extrapolation of the findings regarding directionality or causation. This study was also conducted via online survey and thus is limited to participants who have relatively easy access to the Internet. The self-selected nature of the survey participants and recruitment through advocacy/support groups may lead to a study population not reflective of the general PWS population. Those who are underserved, socioeconomically disadvantaged or experiencing the highest levels of stress may be underrepresented. Another limitation of the current study was its use of several single item measures, rather than validated questionnaires, to assess caregiver emotional symptoms and relationship impact of caring for a person with PWS. However, there are currently no validated measures of quality of life tailored to PWS, and the goal of this initial exploration was to identify areas of focus for future studies. Future studies should include additional, validated measures of caregiver mental health and quality of life. Findings in the current study are reflective of caregivers who are mostly mothers with children with PWS living at home and living in the United States. Future research should focus on engaging a greater cross section of caregivers assessed in older age groups and in different settings in order to further our understanding of the impact of these factors on caregiver burden in PWS.

## Conclusions

The results of the present study reveal that PWS incurs high caregiver burden and impacts most aspects of caregiver life. PWS caregiver burden data could be considered as an additional dataset reinforcing critical unmet needs for PWS. Consequently, caregivers of children with PWS may represent an underserved clinical population, who warrant attention, assessment and intervention in their own right.
